# Prenatal alcohol exposure among Alaska Native/American Indian infants

**DOI:** 10.3402/ijch.v72i0.20973

**Published:** 2013-08-05

**Authors:** Burhan A. Khan, Renee F. Robinson, Julia J. Smith, Denise A. Dillard

**Affiliations:** Southcentral Foundation, Anchorage, AK, USA

**Keywords:** prenatal alcohol exposure, fetal alcohol syndrome, fetal alcohol spectrum disorder, Alaska Native/American Indian infants

## Abstract

**Background:**

Recent reports indicate a decline in rates of Fetal Alcohol Syndrome (FAS) among Alaska Native and American Indian (AN/AI) infants. Nevertheless, AN/AI infants remain disproportionately impacted by the effects of prenatal alcohol exposure.

**Methods:**

AN/AI pregnant women in their 3rd trimester completed a questionnaire on demographic data and the amount and frequency of their alcohol consumption in the month prior to conception and during pregnancy. Differences across demographics and trimesters were tested with the Chi-square, Fisher's exact or McNemar's test as appropriate.

**Results:**

Of the 125 participants, 56% (n=71) reported no alcohol consumption in the 1st through 3rd trimesters of pregnancy; 30% (n=38) of the 125 participants also reported no alcohol consumption in the month before pregnancy. Of the 43% (n=54) who reported consuming alcohol during pregnancy (1st, 2nd and/or 3rd trimester), most (35%) reported alcohol use only in the 1st trimester. Binge drinking in the 1st or 2nd trimester was reported amongst 20% (n=25) of participants with an additional 18% (n=29) reporting binge drinking in the month prior to pregnancy. Women who reported pre-conception binge drinking were significantly more likely to report binge drinking during their 1st trimester (p<0.0001) and 2nd trimester (p<0.0001). A history of tobacco use (p=0.0403) and cigarette smoking during pregnancy (p<0.0001) were also associated with binge drinking during pregnancy.

**Conclusion:**

Among study participants, reported use of alcohol was primarily limited to pre-conception and the 1st trimester, with a dramatic decrease in the 2nd and 3rd trimesters. Prevention programmes, such as the Alaska FAS Prevention Project, may have contributed to observed decreases in the 2nd and 3rd trimesters. Additional study and focus on pre-conception, the 1st trimester and binge drinking, as well as tobacco use might augment Fetal Alcohol Spectrum Disorder prevention efforts.

Alcohol use during pregnancy, or prenatal alcohol exposure (PAE), is a national concern, as alcohol use can negatively impact a woman's health and can be passed across the placenta to a developing foetus. Alcohol abuse during pregnancy poses risks to the foetus (including poor growth, decreased muscle tone, delayed development, heart defects, physical/structural problems and mental retardation) known as Fetal Alcohol Syndrome (FAS) and Fetal Alcohol Spectrum Disorder (FASD) (1). FAS is the term used to describe growth, mental and physical problems that may occur in an infant when a mother consumes alcohol during pregnancy, whereas FASD is the term used to describe the additional direct and indirect social, physical and emotional effects (2–4).

FAS is one of the most preventable causes of mental retardation in the United States (1, 5). Annual long-term economic and societal costs associated with FAS and FASD are in the billions (2–4, 6). In 2002, Alaska was assessed as having the highest FAS prevalence rates in states using similar surveillance methodologies (1, 7–12). Between 1996 and 1998, FAS prevalence was 15-fold higher in the Alaska Native population than the general Alaska population (13). Though this discrepancy has since decreased, Alaska Native infants still have a disproportionally higher prevalence of FAS with 32 Alaska Native infants with FAS compared to 6 Non-Native Alaskan infants with FAS per 10,000 live births between 2000 and 2002 (14).

Abstinence from alcohol has been recommended for women who are pregnant or may become pregnant. However, based on studies in the general population, prenatal abstinence from alcohol is estimated to be low (<20% of pregnant women are abstinent in the 1st trimester) (15). No “safe” level of alcohol use during pregnancy has been established, and prevalence of alcohol use among pregnant and non-pregnant women of childbearing age continues to be a concern. However, larger amounts of alcohol and binge alcohol drinking (currently defined for women as ≥4 drinks per sitting) appear to be more harmful than smaller amounts of alcohol ingestion (16, 17). Despite increased education and delays in age of conception, drinking behaviors do not appear to have significantly changed (18). During 2001–2005, the highest percentages of pregnant women in the United States reporting any alcohol use were women aged 35–44 years (17.7%) and women with college degrees (14.4%) (19).

Alcohol ingestion in pregnant Alaska Native/American Indian (AN/AI) women is an even greater public health concern than the general US population (13, 20–22). Given high rates of self-reported alcohol use in adults and the high prevalence of FAS and FASD in AN/AI infants, understanding alcohol intake habits of AN/AI pregnant women is vital to develop targeted prevention strategies (1, 7–12). As FASD among infants is a direct result of PAE among pregnant women, there is a need to better identify, document and understand alcohol consumption of AN/AI women during pregnancy (e.g. exposure to alcohol through over-the-counter medications, absolute alcohol consumption and occurrence of binge drinking). In this study, we assess self-reported PAE among AN/AI women.

## Methods

### Setting

Southcentral Foundation's Primary Care Center (SCF-PCC) in Anchorage, Alaska, provides pre-paid primary care services to approximately 45,000 eligible AN/AI people in the urban and remote rural surrounding areas of Anchorage.

### Recruitment

Any AN/AI woman ≥21 years of age, in her 3rd trimester of pregnancy, and eligible for care at the SCF-PCC was eligible to participate in the study.

### Questionnaire

After consent, women were asked to complete a detailed questionnaire on alcohol exposure for each trimester of pregnancy and the month prior to pregnancy. Participants identified both the month in which they found out they were pregnant (received a positive pregnancy test) and the month of their 1st prenatal visit. Based on answers to these questions, the recruiter determined the month prior to pregnancy, 1st trimester, 2nd trimester and 3rd trimester. If consumption of beverages containing alcohol was reported, additional questions about alcohol type, frequency of consumption and amount consumed were asked. Women were also asked about their alcohol consumption during the month before pregnancy and each trimester. The 9 types of alcoholic beverages assessed were beer, malt liquor, wine, sweet wine, fortified wine, wine coolers, hard liquor, mixed drinks and liqueurs. Questions regarding age, height, weight (before pregnancy) and smoking status were also included on the questionnaire.

### Data collection and categorisation

De-identified questionnaire data were entered and verified using QDS 2.5 software (Bethesda, MD). Daily absolute alcohol values were calculated from participants’ responses to type and volume of alcohol by adjusting reported ounces consumed per day to a number of standardised drinks and multiplying by 0.5 ounces of absolute alcohol per standardised drink. For example, 4 ounces of wine represented a standardised drink and thus represented consumption of 0.5 ounces of absolute alcohol. Categorisation of absolute alcohol consumption was adapted from prior studies with <0.01 fluid ounce (fl. oz.) per day, indicating abstinence from alcohol (i.e. <12 drinks a year), 0.01–0.21 fl. oz. per day indicating light drinking (i.e. <3 drinks a week), 0.22–1.00 fl. oz. a day indicating moderate drinking (i.e. 3–14 drinks per week), and >1.00 fl. oz. a day indicating heavy drinking (i.e. >14 drinks per week) (23, 24). At the time of the study, binge drinking was defined as ingesting 5 or more drinks in one sitting, and thus was assessed as such on the questionnaire (16, 25–27). It should be noted that since the time this study was conducted, the definition of female binge drinking has been reduced from ≥5 drinks to ≥4 drinks in one sitting, according to the National Institute of Alcohol Abuse and Alcoholism (NIAAA).

### Data analysis

Statistical analyses were performed using SAS 9.2 software (Cary, NC). Associations with reported drinking were investigated with the Chi-square Test of Proportions or Fisher's Exact Test when appropriate. Associations of reported drinking between time periods were tested with McNemar's Test. P-values <0.05 were considered significant.

## Results

### Demographics

Over the course of the recruiting period, 125 AN/AI pregnant women were enrolled into the study. The average age of participants was 26.8 years of age, with a range of 21–39 years of age.

### Reported drinking during pregnancy

**Fig. 1:**
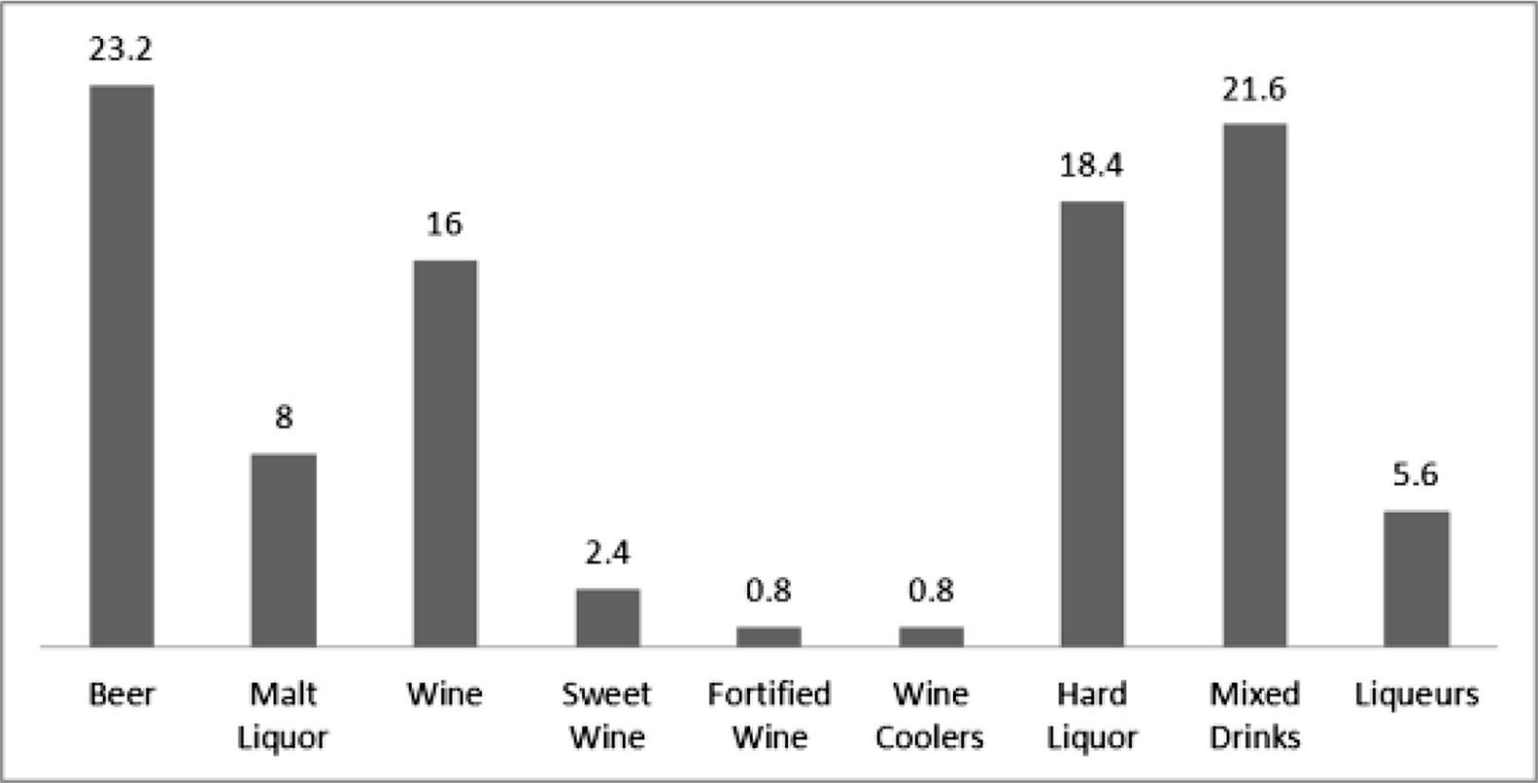
Type of alcohol ingested during pregnancy (percentages of cohort).

Of the 125 participants, 43% (n=54) reported drinking alcoholic beverages during pregnancy (1st, 2nd and/or 3rd trimester), with 35% (n=44) reporting alcohol use in the 1st trimester only. The remaining 8% (n=10) reported alcohol use in time periods other than the 1st trimester. Of the 80 women who reported alcohol use for the month prior to pregnancy, 59% (n=47) reported drinking during pregnancy. Of the 71 women that reported no alcohol use during the 1st, 2nd and 3rd trimesters, 54% (n=38) reported alcohol use in the month prior to pregnancy. Thirty percent (30%) of the total participant pool (n=38) reported no alcohol consumption from the month before pregnancy through the 3rd trimester. The most prevalent types of alcoholic beverages consumed during pregnancy were beer (23.2%), mixed drinks (21.6%), hard liquor (18.4%) and wine (16%) (Fig. 1).

### Absolute alcohol consumption

**Fig. 2:**
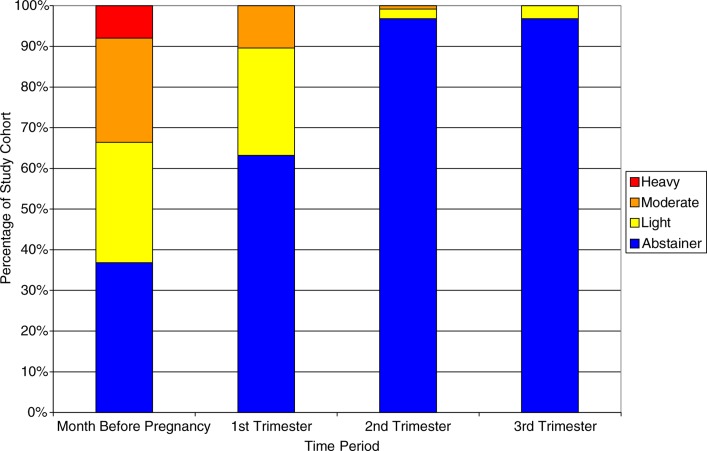
Daily absolute alcohol consumption categorisations by trimester.

Daily reported absolute alcohol consumption was compared by trimester and the month prior to pregnancy (Fig. 2). Over the course of the pregnancy, daily values of absolute alcohol consumption decreased heavily between the 1st and 2nd trimesters, with the majority of participants fitting into the abstinence category for the 2nd and 3rd trimesters. Average daily absolute alcohol consumption decreased over the duration of reporting period from 0.371 fl. oz. (month before) to 0.055 fl. oz. (1st trimester) to 0.004 fl. oz. (2nd trimester) to 0.001 fl. oz. (3rd trimester).

### Binge drinking

Twenty percent (n=25) of participants reported at least 1 occurrence of binge drinking during the 1st or 2nd trimester, with an additional 18% (n=29) reporting binge drinking in the month prior to pregnancy. No women reported binge drinking in the 3rd trimester. Demographics of women reporting binge drinking are detailed in Table I. Although age was not associated with binge drinking during the month before pregnancy (p=0.4288), a higher percentage of women in the youngest age category reporting binge drinking compared to the older categories during pregnancy, though this relationship was not statistically significant (p=0.0544). History of tobacco use (p=0.0403) and smoking tobacco use during pregnancy (p<0.0001) were also associated with binge drinking during pregnancy. However, body mass index was not associated with binge drinking before or during pregnancy. Importantly, women who reported binge drinking during the month before pregnancy were significantly more likely to report binge drinking during their 1st trimester (p<0.0001) and 2nd trimester (p<0.0001, data not shown).

**Table I:** Demographics and predicative comparisons for binge drinking during pregnancy and the month prior to pregnancy

	Month before pregnancy					Pregnancy^a^				
				
	Yes		No			Yes		No		
						
Binge drinking (125 Surveys)	n	%	n	%	p^b^	n	%	n	%	p^b^
**Demographics**										
Age (16 missing)										
21–25 years	22	42.31	30	57.69	0.4288	15	28.85	37	71.15	0.0544
26+ years	19	33.33	38	66.67		7	12.28	50	87.72	
Smoking during pregnancy or month prior (2 missing)										
Yes	36	59.02	25	40.98	**<0.0001**	22	36.07	39	63.93	**<0.0001**
No	14	21.88	50	78.13		3	4.69	61	95.31	
Body Mass Index (27 missing)										
Underweight/normal weight	11	34.38	21	65.63	0.6198	6	18.75	26	81.25	1.0000
Overweight	8	29.63	19	70.37		5	18.52	22	81.48	
Obese	16	41.03	23	58.97		8	20.51	31	79.49	
**Predictive comparisons**										
Binge drinking during 1st trimester										
Yes	20	83.33	4	16.67	**<0.0001**					
No	30	29.70	71	70.30							
Binge drinking during pregnancy^a^										
Yes	21	84.00	4	16.00	**<0.0001**					
No	29	29.00	71	71.00							

a Includes reported drinking in 1st, 2nd and/or 3rd trimester.

b Demographics comparisons used Chi-square test of proportions unless cell counts were too small in which case Fisher's exact test was used. Predictive drinking comparisons used McNemar's Test. Significant p-values in bold.

## Discussion

In our study, self-reported alcohol use among AN/AI women during pregnancy (~50%) continues to be higher than in the general population. Reported drinking was primarily limited to pre-conception and the 1st trimester, with a dramatic decrease in the 2nd and 3rd trimesters. Prevention programmes, such as the Alaska FAS Prevention Project, may have contributed to noticeable decreases, especially in the 2nd and 3rd trimesters; however, alcohol exposure during pre-conception and during the 1st trimester remains high and of concern. Binge drinking pre-conception was also associated with binge drinking in the 2nd trimester and during the entire pregnancy. Thus, additional study focused on pre-conception, the 1st trimester and binge drinking might augment FASD prevention efforts among AN/AI women (7, 10, 15, 28, 29). For instance, providers could be encouraged to routinely discuss childbearing plans with women they serve and then encourage abstinence from alcohol among women as they try to become pregnant or among sexually active women without effective contraception. Such efforts may potentially attenuate alcohol use very early in the 1st trimester when women may not know they are pregnant.

Since all drinks do not contain the same amount of alcohol, data were collected to identify the type and quantity of beverage ingested. In our study, absolute ethanol consumption was quite variable and ranged from 0 to 237.5 fl. oz. per trimester (Fig. 2) and included a variety of drinks (Fig. 1). Efforts to better identify and understand consumption habits of AN/AI women during pregnancy are vital for targeted PAE prevention strategies. Providers should review with pregnant women the risks associated with ingesting alcohol, making note of the risks associated with different types and volumes of drinks (20, 30).

Binge drinking is particularly harmful to foetal brain development (19). In this study, we found significant pre-conception binge drinking, and we found pre-conception binge drinking to be strongly associated with binge drinking during the 1st trimester (Table I). Given the current lowered threshold for binge drinking to ≥4 drinks in 1 sitting, estimates of binge drinking we present may be underestimating the prevalence according to current definitions. Younger women were more likely to binge drink (Table I), suggesting a need for more screening and FAS education of women of childbearing age and during early pregnancy. According to a nationwide, postpartum survey, 42.5% of all Alaskan women having a live birth reported the pregnancy was either mistimed (32.4%) or not planned (10.1%). Contraceptive use among these women was reported at 45.3%. Considering these percentages and the prevalence of pre-conception binge drinking in our cohort, healthcare providers should encourage abstinence from alcohol among Alaska Native women who may become pregnant, whether using contraception or sexually active and not using contraception. Efforts can be targeted at younger women, as they were more likely to continue binge drinking into their pregnancy.

Future research to identify Alaska Native women's views regarding pregnancy may help establish appropriate pregnancy planning programmes and further understanding of social and/or cultural characteristics affecting pregnancy. Chang et al. has developed and tested a 4-item alcohol exposure screening tool proven to be more sensitive during pregnancy than typical obstetric staff assessment in ethnically diverse populations (29). Based on our data, this screening tool may be useful to identify women at increased risk in the AN/AI population. In addition, the questionnaire used did not differentiate between alcohol exposure very early in the 1st trimester when women may not know they are pregnant versus alcohol exposure later in the 1st trimester. Furthermore, as women were recruited in the 3rd trimester, recall of alcohol ingestion in the 1st and 2nd trimesters may not have been accurate. Another limitation to our study was sample size. Our study achieved a quarter of the original recruitment goal. A prenatal tobacco exposure study recruiting in parallel with this study enrolled 3-times as many participants. This observation suggests a reluctance of AN/AI pregnant women to enrol into prenatal alcohol-related studies. Social stigma associated with drinking during pregnancy may have been a barrier in achieving the original recruitment goal and thus attaining an even more representative participant population of pregnant AN/AI women. Finally, another limitation to our study was the age of the respondents. Based on the legal drinking age, we decided to look at women aged 21 years and older. This may underestimate the impact of underage drinking on the prevalence of FAS in the AN/AI community.
